# Koumiss as a Remedial Agent — Report of Cases

**Published:** 1875-05

**Authors:** R. L. Leonard

**Affiliations:** Chicago


Article II.

### KOUMISS AS A REMEDIAL AGENT — REPORT OF CASES.

By R. L. LEONARD, M.D., Chicago.

While I trust the profession will not think me too sanguine in estimating the favorable results of a new remedial agent, it has been my fortune to have an early experience in the use of koumiss (fermented mare's milk) and the effect of its use has been such that I deem it proper to call the attention of the profession to a few cases which have been materially benefited by its use.

The first case, P., a babe, born at full term, and apparently healthy, weighing eight pounds. The mother's breasts secreted little or no milk, and from its birth the child was obliged to subsist upon cow's milk. For a few days it seemed to thrive, but the fifth day it vomited a good deal; an occasional ten-grain powder of the subnitrate of bismuth was ordered. On the seventh day my little patient was no better, and I gave aqua calcis, adding it to the cow's milk. When I called on the eighth day it was reported to be vomiting two-thirds of all the milk taken. I ordered the nurse to give it condensed milk instead of the new cow's milk, and no medicines, and when I called on the ninth day, found that there was little or no vomiting, but on the tenth day the vomiting had increased considerably, and there was a very loose condition of the bowels. Subnitrate of bismuth was ordered in ten-grain doses every four hours, and for a day or two the child was better, but on the fourteenth day when I called, was reported much worse. The bismuth, which had been discontinued, partially, in view of the marked improvement noticeable at my last visit, was now resumed, and the dose increased to fifteen grains, but in spite of treatment, on the fifteenth day the babe retained but little of either milk or medicine, was constantly worrying, and its extreme emaciation induced me to obtain its weight, which I found to be seven pounds. On the eighteenth day the mother reported that it had vomited everything (milk and medicine) for forty-eight hours, and it seemed about dying from actual starvation, and my opinion was that it could not live another twenty-four hours unless something nourishing was retained.

In this extremity it occurred to me to try koumiss. Acting upon this idea I obtained a quart bottle of medium koumiss, and returning, had the nursing bottle filled with two-thirds koumiss and one-third hot water, and gave to the starving babe; to my utter astonishment it took the koumiss with an evident relish, emptied the bottle, and equally astonishing to me was the fact that it was retained. I ordered the mother to give all the babe would take, if retained, and on calling the next day found that it had used the entire quart, and the bowels were moving at intervals of about six hours.

On the 3rd day after commencing the use of koumiss in this case, a slight diarrhœa manifested itself, which disappeared in twenty-four hours without treatment. The babe had all the koumiss it would take from the first, improvement continued to be rapid, and at time of writing the child has used twenty-four gallons of koumiss or an average of about three pints per day, is fat and healthy as a child can be at the age of fourteen weeks, has gone with its mother to her home in Nebraska, and in a letter received to-day she tells me that it has now been fed upon cow's milk for a week and is doing well.

The second case, Mrs. B., a lady of American birth, aged 36 years, who had been debilitated by typhoid fever, in which the stomach was quite irritable, and although the disease had a week previous passed its acme, still but little nourishment could be given on account of this excessive irritability. Koumiss was ordered in its purity, and was acceptable to the stomach ; the recovery of strength was rapid, and the koumiss was discontinued in two weeks.

The third case, Mrs. O., an American lady, aged 43, who had been afflicted with dyspepsia for many years, and for the past ten of which her diet had been confined almost exclusively to oatmeal and her drink to very weak tea, as these were the only things that would remain unvomited. She had, as such patients usually do, visited physicians of eminence, and was during the past year very feeble and confined to her bed full half the time, simply because she was too weak to sit up. I had used koumiss in these other cases of irritable stomach and extreme emaciation and thought it advisable to try it in her case, so ordered it taken gradually for a day or two, then all she could take, to the exclusion of other food and drink. In two weeks she was much improved, so much so that she was not only able to sit up, but to take a broom and sweep the house, an exercise she had been totally incapacitated for during the whole of the last seventeen years. After three weeks, she was able to go for her own koumiss, walking a distance of one and a half miles on that mission every few days, and at present, after two months' use of the remedy, has so far regained her digestive power that she requires but little koumiss and can eat beefsteak and partake of the usual diet of her family table.

The fourth case, J., is a babe, aged six weeks, at the time of my first visit, which was depending upon the bottle, the mother having no milk in the breasts.

When I called, the babe, a little puny thing, seemed as if it were gasping for its last breath; it was extremely emaciated, and for twenty four hours up to that time had been unable to nurse the bottle, and had received an occasional drachm of milk from a teaspoon; the bowels were moving every hour, and it had retained scarcely anything for a day or two. I had some doubt as to whether the babe would live until the messenger could procure koumiss, let alone being benefited by it. A spoonful was put into its mouth which was with difficulty swallowed, soon it was followed by another, and so on at short intervals for four hours, at the expiration of which time it took the koumiss from the bottle, and from the very moment of the administration of the first spoonful to the present time, a period of nearly two weeks, its improvement has been exceedingly rapid, and it is now quite strong and apparently healthy.

I might mention another case, in which Mrs. S., a lady aged about 25 years, debilitated by a miscarriage which was accompanied by excessive hæmorrhage and succeeded by a three months' illness which left her in an extremely anæmic condition, which refused to yield to quinine, iron, strychnia and other tonics, that upon the introduction of koumiss to the treatment of the case, improved rapidly, and in three weeks so far recovered as to be discharged cured.

It is my candid belief that in koumiss the profession has a valuable acquisition to the list of remedies, and while I have dwelt in detail, upon the report of these cases, selected from my experience during the last three months with about fifty gallons of medium koumiss, I am satisfied that my success is not that kind which is attainable only in the hands of the writer, but may be duplicated by my professional brethren who have cases of extreme debility or emaciation to deal with, particularly where the stomach is in an irritable condition without active organic disease; and trusting that the report of these cases may be of service to the readers of the JOURNAL, I submit it to their consideration.

